# Contraception access during the COVID-19 pandemic

**DOI:** 10.1186/s40834-020-00114-9

**Published:** 2020-10-08

**Authors:** Jasmine Aly, Kristin O. Haeger, Alicia Y. Christy, Amanda M. Johnson

**Affiliations:** 1 Program in Reproductive Endocrinology and Gynecology, NICHD, NIH, Bethesda, Maryland USA; 2 Department of Veterans Affairs, Veterans Health Administration, Women’s Health Services, 810 Vermont Ave., NW, Washington, DC, 20420 USA

**Keywords:** Contraception, Access, COVID-19, Pandemic

## Epidemiology

On December 8, 2019, the first case of coronavirus disease 2019 (COVID-19) was reported in Wuhan, China [1]. Chinese health authorities notified the World Health Organization (WHO) on December 31, 2019, and by January 30, 2020, WHO designated the outbreak as a Public Health Emergency of International Concern [2]. By June 10, 2020, over 7 million confirmed cases and over 400,000 deaths had been recorded across 213 countries and territories [3].

In outbreaks, epidemics, and pandemics, epidemiologists aim to quantify the spread of a disease within a population across space and time. In addition, epidemiologists aim to quantify the rate of disease transmission. This information is then used to inform prevention and mitigation strategies. Although aggressive prevention strategies may be disruptive and costly, such measures may ultimately reduce the burden of morbidity and mortality within a population, as has been demonstrated in previous pandemics, such as the 1918–1920 influenza and the 2009 influenza A (H1N1) pandemics [4, 5].

The first tier of response is containment to prevent the spread of disease before it has a chance to take hold in the community [6]. This may include contact tracing, surveillance in the community through widespread testing, and quarantine measures. However, once a disease has spread through the community, the second tier, mitigation strategies, are necessary to reduce transmission. Interventions include social distancing measures; closure of schools, workplaces, and community facilities; travel restrictions; and individual-level hygiene measures, such as wearing a mask and washing hands [6]. Without mitigation efforts in place, healthcare systems risk being stretched beyond capacity in, for example, intensive care unit (ICU) beds, personal protective equipment (PPE), and ventilators for treating patients with COVID-19. This is why countries who were in the mitigation phase of the pandemic conducted communication campaigns imploring individuals to engage in behaviors to “flatten the curve.” Beyond the mitigation tier, state-level actors may put lockdowns in place to further curb transmission.

## Scope of the problem

### Asia

Countries across Asia were some of the first to experience the outbreak of COVID-19. Many had already had previous experiences dealing with epidemics, including severe acute respiratory syndrome (SARS) from 2002 to 2003, H1N1 flu in 2009, and Middle East Respiratory Syndrome (MERS) in 2014, 2015 and 2018 [7]. Such experiences had prepared governments to respond and made their populations more receptive to restrictive public health measures. Some entities, including South Korea, Mongolia, Hong Kong, and Singapore, initially succeeded in containing the virus through aggressive preemptive measures: transparency in communication, ubiquitous testing, strict quarantine, and thorough disinfectant protocols [7, 8]. South Korea used such measures without ever putting a lockdown in place. After failures in communication during the MERS epidemic in 2015, new standard operating procedures were put in place. By the time COVID-19 arrived, Koreans were willing to forego privacy protections in exchange for transparency about the spread of the virus. In other countries in Asia, community transmission of COVID-19 negated containment measures. China, Pakistan, and India all implemented abrupt lockdowns in an effort to mitigate the spread of the virus.

Overall, there were 1,462,196 cases and 37,081 deaths from COVID-19 documented in Asia as of June 10, 2020 [3]. India had the greatest burden of disease, with 287,155 cases and 8107 deaths. In a modified susceptible-exposed-infectious-recovered (SEIR) model by the University of California, Los Angeles (UCLA), it was projected that India will have 558,832 cases (95% confidence interval (CI): 391,731–1,031,743) and 20,792 deaths (95% CI: 16,039–34,705) by September 16, 2020, assuming no changes in public health interventions and that infected individuals have life-long immunity after recovery [9]. The Los Alamos National Laboratory (LANL), another statistical model assuming that current interventions will stay in place, forecasted that by July 19, there will be 714,000 cases (90% CI: 439,000–1,440,000) and 19,600 total deaths (90% CI: 12,000–40,300) in India [10]. Limitations of the models include caveats that confirmed deaths and cases are underestimates based only on symptomatic individuals, case definitions may change, data may be skewed by differences in reporting by region or public health department, and seasonality is not figured in. As of June 10, 2020, Mongolia, Vietnam, Cambodia, and Bhutan have each had fewer than 500 cases and no deaths, demonstrating success in containment and mitigation strategies.

### Europe

From China, Italy was the next hardest hit country to experience the global pandemic. Countries across Europe had varied strategies to deal with the novel coronavirus. Sweden eschewed a lock-down and was subsequently criticized for its relatively high mortality rate, 47.5 deaths per 100,000 people as of June 10, 2020 [3]. The United Kingdom loosely implemented a containment strategy and considered attaining herd immunity before trying mitigation strategies as a last resort [11]. In contrast, Greece, like several Asian countries who had also weathered previous epidemics and a financial crisis in recent years, used aggressive measures to contain COVID-19; as of June 10, 2020, it had one of the lowest rates of mortality in all of Europe, with 1.8 deaths per 100,000 people [3].

As of June 10, 2020, Europe had 2,139,738 cases and 180,941 deaths [3]. Six weeks ahead, the LANL model predicted 241,000 cases (90% CI: 237,000–248,000) and 34,600 total deaths (90% CI: 34,100–35,500) for July 17, 2020 in Italy [10]. Three months out, on September 16, 2020, the UCLA model projects 251,481 cases (95% CI: 237,094–390,611) and 35,785 deaths (95% CI: 37,822–35,310) [9].

### US

In 1951, the Communicable Disease Center, now known as the Centers for Disease Control and Prevention (CDC) established the Epidemic Intelligence Service (EIS) in Atlanta, Georgia. *The CDC Field Epidemiology Manual*, a book for EIS officers, provided clear guidance on not only how to handle an epidemic from a public health perspective, but also how to gain trust and buy-in from the public. Emphasis was placed on communication, with instructions to express empathy and explain both what is known and unknown [12]. The manual also made clear that communication should come from a single trusted source, a scientist, so that the public would not worry about whether messaging was based on science or politics. A Single Overriding Health Communication Objective, or SOHCO (pronounced “sock-O”) should be simple and easy to remember, the manual instructed. When the first case of COVID-19 was detected in the US on January 21, 2020, the local health department in Seattle, Washington followed the CDC playbook, the same one that had been used in the H1N1 pandemic of 2009 [2, 13]. A catchy SOHCO was developed and repeated: “more hand washing, less face touching” [13].

However, the US failed to contain the virus and community transmission of COVID-19 took hold. As of June 10, 2020, the US had 2,065,493 cases and 115,126 deaths [3]. Six weeks out, on July 17, 2020, the US was predicted to have 2,550,000 cases (90% CI: 2,240,000–3,110,000) and 135,000 total deaths (90% CI: 123,000–161,000) by the LANL model [10]. By September 16, 2020, the UCLA model forecasted 2,646,472 cases (95% CI: 2,441,012–3,027,482) and 133,367 deaths (95% CI: 130,537–137,503).

Instead of one single pandemic across the country, there were a patchwork of mini-outbreaks, with heterogenous populations and variable underlying comorbidities, all undergirded by systemic racism that directly contributed to health disparities [14]. US states that did not expand the federal and state insurance system for low-income Americans, MEDICAID, experienced a disproportionate share of hospital closures since the Affordable Care Act was implemented in 2015, especially in Southern and rural areas [15]. Efforts at mitigation varied across the country. Lacking coherent federal guidance, states implemented social distancing measures in a piecemeal fashion, thus ending up with the largest burden of COVID-19-related morbidity and mortality in the world.

### Africa

Much like countries in Asia, many countries in Africa had ample experience with previous epidemics: Ebola, cholera, tuberculosis, yellow fever, malaria, and HIV/AIDS. They also had the previous experience of EIS officers from CDC providing technical assistance during previous public health emergencies [16]. By the time COVID-19 emerged, countries bordering the Democratic Republic of Congo, including Rwanda, Burundi, South Sudan, and Uganda were able to nimbly pivot from protocols and rapid response teams used for Ebola to prevent COVID-19 from reaching epidemic proportions [17]. Like Mongolia, countries shut down their borders before a single case was detected [17]. Rwanda responded not unlike South Korea, using contact tracing and isolation; Uganda carried out widespread testing, testing a random sample of 20,000 people; surveillance was completed on the 5 million residents of the capital of Ethiopia to establish medical and travel histories [17]. In one country after another, countries demonstrated that they had learned from CDC’s previous outreach efforts and were able to independently replicate interventions to keep people safe from COVID-19.

As of June 10, 2020, there were 212,003 cases and 5717 deaths recorded in Africa [3]. South Africa has the highest burden of COVID-19, with 55,421 cases and 1210 deaths. Six weeks out, the LANL model predicts 158,000 cases (90% CI: 90,400–389,000) and 3400 total deaths (90% CI: 1900–8500) by July 19, 2020 [10]. Three months out, UCLA forecasted 229,990 cases (95% CI: 124,841–409,707) and 11,436 deaths (95% CI: 5833–21,283) by September 16, 2020 [9]. Countries in Sub-Saharan Africa including Rwanda, Malawi, Zimbabwe, and Burundi each had fewer than 500 cases of COVID-19 as of June 10, 2020.

### Global

With more than 7 million cases of COVID-19 worldwide, the health care emphasis has shifted to focus on the apportionment of resources, prevention of viral spread, emergent medical care, and the development of novel treatments and vaccines [3]. The unfortunate byproduct of this shift is the de-prioritization of other essential health care services such as access to contraception. The consequence of limited access to contraception is evidenced by historic and current data [18–21]. Research conducted by the United Nations sexual and reproductive health agency (UNFPA) projects that more than 47 million women could lose access to contraception leading to 7 million unintended pregnancies as a result of the COVID-19 crisis [22]. The devastating result of unintended pregnancy is an increase in maternal and neonatal morbidity and mortality. Data from the Ebola virus outbreak in Western Africa shows service disruption in maternal and newborn care contributed to an estimated 3600 maternal deaths, neonatal deaths, and stillbirths. This is nearly equal to the number of deaths caused by the Ebola virus itself [21]. Disruption in contraception access also results in an increase in unsafe abortions, miscarriage, pregnancy complications, transmission of HIV and other sexually transmitted infections, as well as increased incidence of post-traumatic stress disorder, depression, suicide, and intimate partner violence [18, 21, 22]. These consequences disproportionally affect developing countries and marginalized groups in the United States, further increasing national and international healthcare disparities. This review will examine the impact of access to contraception in developed and developing countries, highlighting barriers to contraception access. The secondary aim is to provide strategies to mitigate the negative impacts of limited contraception access.

## Impact of limited contraception availability in undeveloped countries

**Fig. 1:**
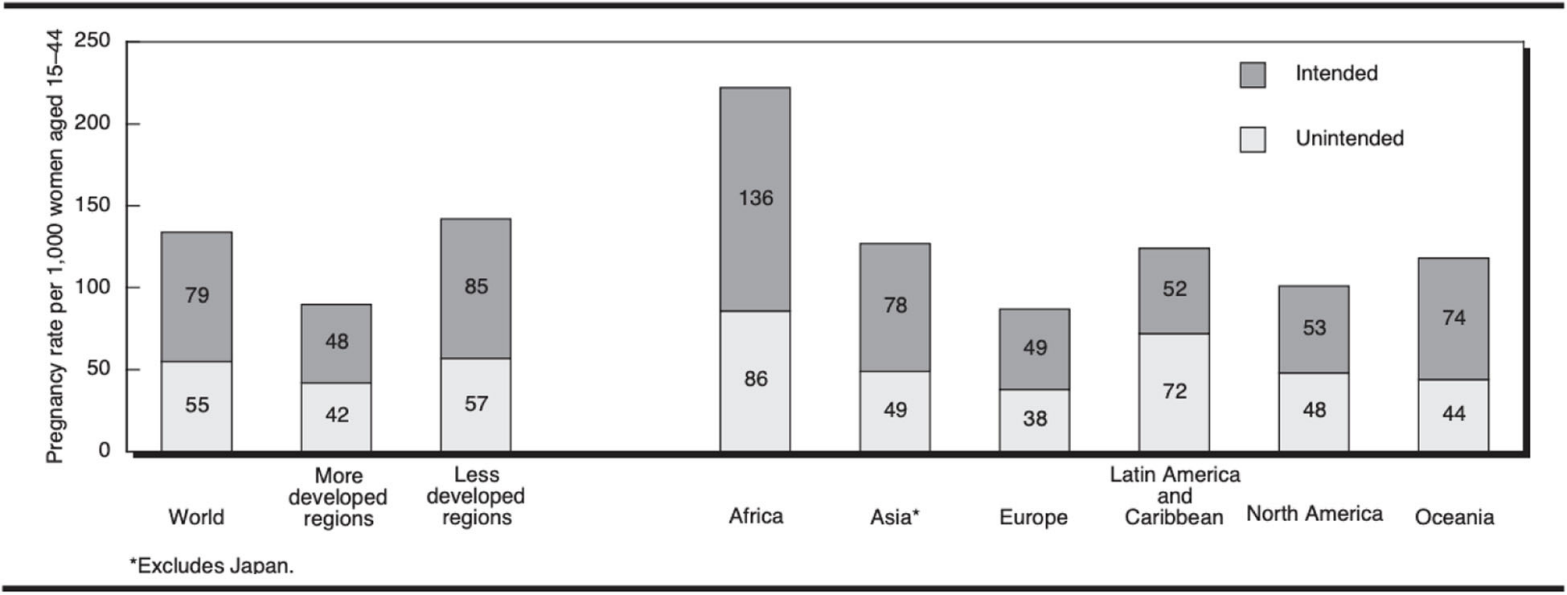
Rates of Unintended and Intended Pregnancy, worldwide and by region, 2008. (Singh et al. [23])

Data from a study by Singh et al., emphasizes the disproportionate rate of unintended pregnancy in developing countries, which preceded COVID-19 [23]. The data shows the unintended pregnancy rate is particularly high in developing regions, and especially in sub-Saharan Africa (Fig. 1) [23]. In 2008, an estimated 140 million women in the developing world who would prefer to delay or cease childbearing were not using contraception, and an additional 75 million were using traditional methods that have high failure rates [23, 24]. These data suggest, even prior to the COVID-19 crisis, a high rate of women with unmet need for contraception. With the additional strain imposed by COVID-19, populations within an already burdened healthcare system will be most vulnerable.

Data also shows that half of all women worldwide resolve unwanted pregnancies with induced abortion [23]. Pre-COVID-19 data from WHO shows that unsafe abortion is the cause of one in seven maternal deaths and results in the hospitalization of approximately 5 million women annually in developing countries [25]. With limited access to contraception during the COVID-19 pandemic, the incidence of unsafe abortions has the potential to increase exponentially in developing countries. Moreover, due to reallocation of inpatient resources related to the pandemic, hospitals may not be able to accommodate the increased volume of women suffering life-threatening consequences of unsafe abortions. A study by Riley et al., used mathematical modeling to project the impact of COVID-19 on reproductive health in low- and middle-income countries by using the most recent contraceptive data for each individual country. In a model assuming that just 10% of safe abortions would be converted to unsafe abortions due to restricted access related to COVID-19, an additional 3.3 million unsafe abortions would occur, resulting in 1000 maternal deaths (Table 1) [26].

**Table 1:** Potential annual impacts of a 10% proportional decline in use of sexual and reproductive health care services resulting from COVID-19–related disruptions in 132 low- and middle-income countries. Source: Riley et al. [26]

Disruption in essential sexual and reproductive health care	Impact
10% decline in use of short- and long-acting reversible contraceptives	48,558,000 additional women with an unmet need for modern contraceptives
	15,401,000 additional unintended pregnancies
10% decline in service coverage of essential pregnancy-related and newborn care^a^	1,745,000 additional women experiencing major obstetric complications without care
	28,000 additional maternal deaths
	2,591,000 additional newborns experiencing major complications without care
	168,000 additional newborn deaths
10% shift in abortions from safe to unsafe^b^	3,325,000 additional unsafe abortions
	1,000 additional maternal deaths

Notes: Service changes are presumed to be the average change over a year, and impacts are on an annual basis

^a^The 10% reduction in service coverage encompasses changes in access for some interventions (e.g., delivery in a facility) and changes in the content or quality of care for others (e.g., provision of magnesium sulfate for eclampsia treatment)

^b^Unsafe abortions are those performed by persons lacking the necessary skills, or in an environment that does not conform to minimal medical standards, or both

**Fig. 2:**
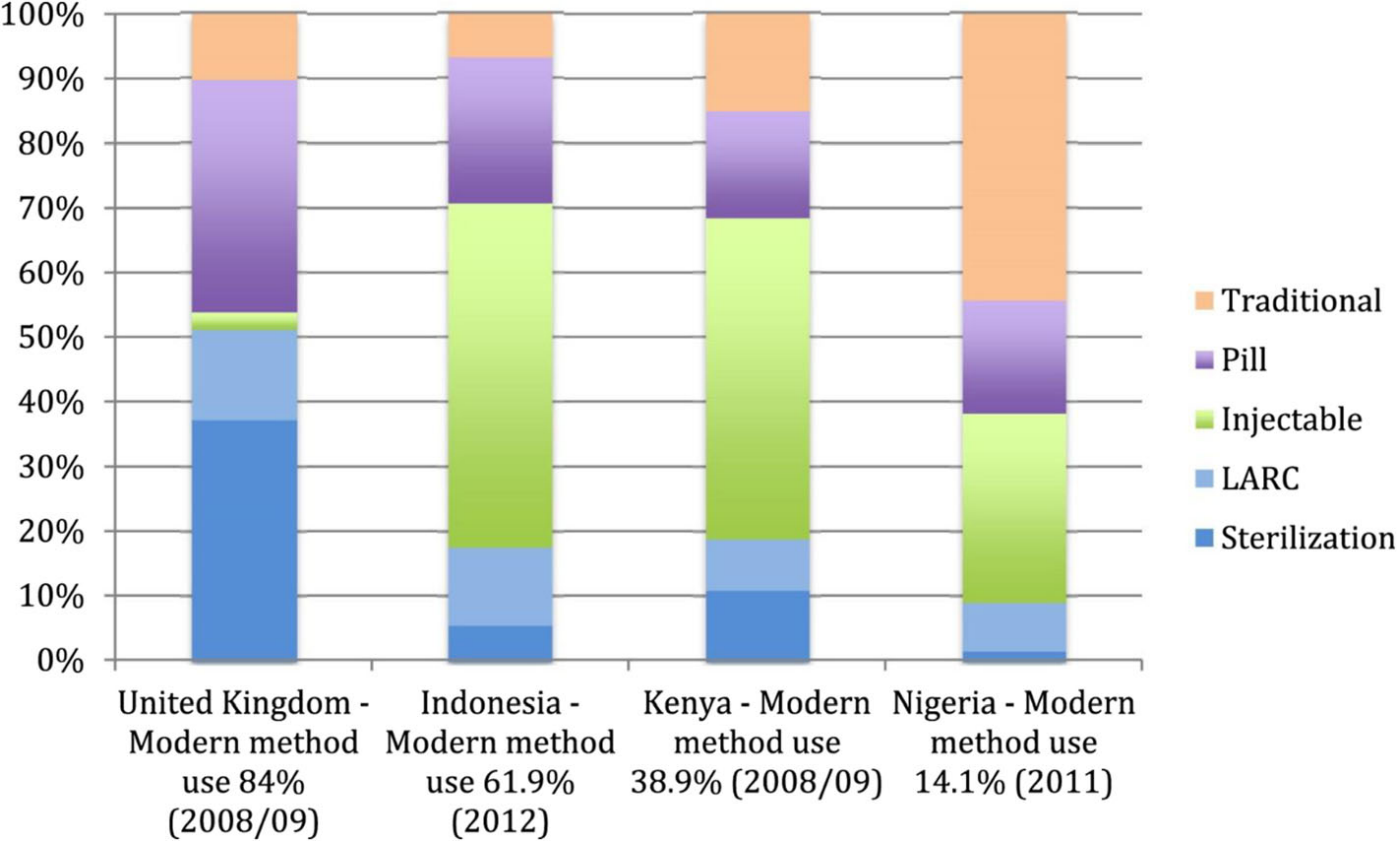
Percent of women using each indicated method of contraception by country. (United Nations, Department of Economic and Social Affairs, Population Division. 2013)

Long-acting reversible contraceptives (LARC) are methods of contraception that are effective for an extended period without requiring user action. They include injections, intrauterine devices (IUDs), and subdermal contraceptive implants. As their efficacy is not reliant on patient compliance, LARCs are the most effective reversible methods, with both typical use and perfect use failure rate of less than 1% per year [27]. LARC usage has played a critical role in reducing maternal morbidity worldwide [28, 29]. In many developing nations, LARCs have become one of the most commonly used methods of contraception (Fig. 2). As these methods require a visit with a medical practitioner to initiate usage, their usage has been curtailed during the COVID-19 pandemic. Based on the modeling study by Riley et al., it is estimated that a 10% decline in the use of short and long acting reversible contraceptive measures due to reduced access would result in an additional 59 million women with unmet need for contraception and an additional 15 million unintended pregnancies in developing countries over the course of one year (Table 1) [26].

Finally, women who do become pregnant during the pandemic are more likely to develop major complications and, due to strained hospital services, may not receive the appropriate care required to address these complications. The model by Riley et al., projects that even with a modest decline of 10% coverage in pregnancy related and newborn health care would have disastrous implications for the lives of women and their newborns. An additional 1.7 million women who give birth and 2.6 million newborns would experience major complication but would not have access to appropriate care during the COVID-19 pandemic, which would result in an additional 28,000 maternal deaths and 168,000 newborn deaths in developing countries (Fig. 2) [26].

## Barriers to access to contraception in developing countries during the COVID-19 pandemic

One of primary barriers to access is a shortage of contraceptive medications and devices, as a result of supply chain disruption. This is a worldwide problem; however, it is more pronounced in developing countries [19]. In India, the government has placed a limit on the export of 26 pharmaceutical ingredients and medications. Among the restricted drugs is the hormone progesterone, which is used in the contraceptive pill and IUDs. As India is one of the world’s largest producers and exporters of drugs, the global distribution of contraceptive pills and IUDs is at risk of disruption [30, 31]. The world’s largest condom manufacturer (Malaysia’s Karex Bhd), which produces one in every five condoms in the world, was forced to close in March, thereby limiting the export of condoms [32]. Closure of borders and travel restrictions also affect the distribution of contraceptive medications and devices [33]. A report from the UN regarding the impact of COVID-19 on reproductive health states that national members of the International Planned Parenthood Federation (IPPF) also face commodity and supply shortages, with 59 members reporting delays in moving goods within countries, and 29 facing a shortage of contraceptives [26].

Contraception provision is considered by some policy makers and directors of medical institutions to be a nonessential activity, and therefore many clinics have been ordered to halt operations. In clinics that have remained open, appointments for contraception counseling and contraceptive administration may have been de-prioritized and re-scheduled. In addition to physical clinic closures, attendance is further affected by patients’ fears of contracting COVID-19, and limitation of transportation services in certain countries. Reports from reproductive health stakeholders in a number of countries report large decreases in numbers of women attending reproductive health clinics [26]. A survey from the IPPF reported the closure of 5633 static and mobile clinics and community-based care outlets across 64 countries, which is approximately 14% of the total IPPF service delivery points in 2019 [26]. According to a United Nations report, South Asia and Africa have been most affected with South Asian partners reporting more than 1872 clinics and other outlets closed, and African partners reporting the closure of 447 mobile clinics [30]. However, the impact is worldwide, with Pakistan, El Salvador, Zambia, Sudan, Colombia, Malaysia, Uganda, Ghana, Germany, Zimbabwe, and Sri Lanka all reporting more than 100 closures of clinics and/or community-based service outlets. The UN report also states that closures have led to severe cuts in sexual and reproductive healthcare services. Specifically, 44 national members reported the scaling down of HIV testing, 41 members reported the scaling down of contraceptive care service, 36 members reported the scaling down services regarding gender-based violence, and 23 members reported the reduction in the availability of abortion care [30].

Another factor affecting contraceptive provision is the availability of health care workers to physically staff clinics. A study regarding the impact of COVID-19 in Kenya, Uganda, and Tanzania reports that prior to the pandemic, there was a longstanding shortage of health professionals, in addition to overloaded health facilities [33]. As such, midwives play a pivotal role in care provision, by delivering contraception and obstetric care to women and families in remote areas or overwhelmed systems [34]. According to the study, since the onset of the COVID-19 pandemic, they have seen an increased need for midwifery care, likely due to the increase in unintended pregnancies. However, a study from WHO states that midwives in all three countries are fearful and hesitant to provide care due to inadequate availability of personal protective equipment (PPE) [35]. In addition, there are country-specific barriers limiting mobility of midwives. For example, in Uganda, travel by private car was banned, limiting the capability of midwives to travel to and from work and/or patient’s homes. Plagues of locusts and flooding also causing significant problems. Women in rural areas, who heavily rely on the services of midwives and mid-level providers, are particularly at risk of negatively being affected by limited provisions [36].

## Barriers to access of contraception in developed countries during the COVID-19 pandemic

In the US, travel restrictions, quarantine measures, additional caregiving responsibilities, fear of exposure to the virus, and fewer appointments due to reduced provider and staff availability are barriers to accessing contraception. Access to abortion during the COVID-19 pandemic has been specifically tumultuous for Americans. In the US, one in four women will use abortion services by the age of 45 [37]. Despite statements in support of continued abortion care by the American College of Obstetrics and Gynecologists (ACOG) and the American Medical Association (AMA), thirteen states (Alaska, Alabama, Iowa, Indiana, Kentucky, Louisiana, Mississippi, Ohio, Oklahoma, Texas, Tennessee, Utah, and West Virginia) have attempted to halt abortion services by deeming abortions “non-essential” or “elective” procedures [38]. With fewer providers available, patients, especially those seeking later abortion, may have to travel further for care. However, in the US, travel by air, bus, and train remains limited. Additionally, in many states, police have set up border checks in order to screen out-of-state drivers for COVID-19, which may result in a two-week quarantine. Anxieties, at baseline, regarding interacting with police are magnified for people of color and those who are undocumented. Anxieties are further compounded by the emotional injury sustained by minorities in the recent wake of the killing of George Floyd, and the many other minorities who have suffered injury or death at the hands of the police. In the US, minorities are already at higher risk of poorer health outcomes and decreased access to health care services. The limitation of contraception access, as a result of the COVID-19 pandemic, will exacerbate racial and ethnic disparities which are pervasive in women’s health.

## Selected strategies for improving contraception access during the COVID-19 pandemic

ACOG recommends the implementation of telehealth visits to screen, counsel, prescribe, and manage complications related to oral contraceptives. They recommend refilling contraceptives for the full year, and providing advance prescriptions for emergency contraception, particularly for ulipristal acetate. With regard to LARCs, they recommend continuing to offer insertion of IUDs and contraceptive implants, and permanent contraception where possible. If LARC methods are unavailable, they recommend prescribing oral contraceptives as a bridge to delayed insertion. Importantly, they also recommend postponing routine LARC removals, if possible, due to the demonstrated effectiveness of extended use beyond the labeled duration. Clinical trial data supports extended use of LARC devices beyond approved durations as follows: the copper T380A remains effective for 12 years, the 52 mg levonorgestrel IUD for 7 years, and the etonogestrel implant for 5 years of use. ACOG also supports women taking advantage of pharmacist-prescribed over-the-counter hormonal contraception programs in states where this practice is in place [39].

With regard to abortion, ACOG, along with numerous health organizations including American Society for Reproductive Medicine (ASRM) and the Society of Family Planning (SFM), released a statement in March stating, “Abortion is an essential component of comprehensive health care. It is also a time-sensitive service for which a delay of several weeks, or in some cases days, may increase the risks or potentially make it completely inaccessible. The consequences of being unable to obtain an abortion profoundly impact a person’s life, health, and well-being… [We] do not support COVID-19 responses that cancel or delay abortion procedures. Community-based and hospital-based clinicians should consider collaboration to ensure abortion access is not compromised during this time.” [40] They also recommend a “no-test” protocol, which allows for appropriate patients at < 77 days of gestation to have access evidence-based, safe medication abortion without in-person visits. The treatment package includes mifepristone, misoprostol, ibuprofen, and/or post-abortion contraception [41].

SFM recommendations align with most ACOG recommendations with the additional mention of some measures. SFM recommends not withholding oral contraceptives for lack of blood pressure or BMI documentation. Specifically, if a patient does not have a documented blood pressure, they recommend advising the patient to check their blood pressure using a purchased cuff at home. However, if one is not available, they recommend prescribing contraceptives after counseling patients of the risks. With regard to depot-medroxyprogesterone acetate (DMPA) injections, they recommend considering a prescription for the subcutaneous formulation, which can be self-administered. They also recommend maintaining access to postpartum tubal ligation [42].

WHO recommends countries develop innovative strategies to ensure as many eligible people as possible can access information and contraception during this period by increasing the use of mobile phones and digital technologies [43]. In addition to expanding availability of contraceptive resources in healthcare facilities, they recommend increasing information and access at pharmacies, drug shops, online platforms, and other outlets. With regard to emergency contraception, they support developing plans to increase access to emergency post-coital contraception, including consideration of over-the-counter provision. They recommend enabling access to contraception for women and girls in the immediate post-partum and post-abortion periods. Finally, they encourage health care workers to provide contraceptive information and services as per national guidelines to the full extent possible, particularly in areas where pregnancy poses a high risk to health [43].

## Development of a response toolkit

The ability of a region to maintain delivery of contraception during the COVID-19 pandemic depends on the baseline stability of the health system in the country, the baseline incidence of unintended pregnancy, the local burden of COVID-19 disease, and access to government programs which may provide collaborative aid. It is clear that mobilization to procure contraceptive resources will be required at multiple levels, including global women’s health organizations, governmental leadership, large and small hospital systems, federally funded and privately-owned clinics, and rural clinics. To organize these factions, we suggest each country or region develop a dedicated team with representatives or liaisons from each of the aforementioned entities. The goal of the task force will be to develop a region-specific approach to maintain equitable access to high-quality contraceptive services throughout the COVID-19 crisis, which can be applied in future conditions of regional crisis.

First, the taskforce should develop an advocacy plan aimed at garnering support from country leadership in recognition of contraceptive access as an essential medical service. Simultaneously, an accounting of the national and subnational levels of available resources should be compiled. The task force should emphasize the unique resources required for their respective vulnerable and marginalized populations, as they will likely be most acutely affected. Gaps in access to contraception should be identified. Efforts should be made to bolster current supply chains of contraception medications and devices in anticipation of a shortage. WHO has suggested a number of key actions that can be taken to secure additional suppliers and streamline supply chain distribution (Table 2). Government liaisons should facilitate access to women’s health clinics by reforming regulations which limit the transportation to and from clinics. Governmental liaisons should incentivize healthcare workers to staff women’s health clinics by providing adequate PPE and compensation. Hospital systems liaisons and clinic liaisons should prepare to meet the substantially increased needs of patients presenting with complications from unsafe abortions, miscarriage, pregnancy complications, increased transmission of HIV and other sexually transmitted infections, as well as the increased incidence of post-traumatic stress disorder, depression, suicide, and intimate partner violence. Hospital and clinic liaisons can apply the aforementioned suggested strategies by ACOG, SFM, IPPF, and WHO to develop specific plans for preparedness and response support. A well-organized and prepared health system, which has the capacity to support access to contraceptive services during the COVID-19 crisis, will reduce unintended pregnancies, thereby mitigating the profound increase in maternal morbidity and mortality that would ensue as a result of limited contraceptive access.

**Table 2:** Key Actions to Strengthen Supply Chains. WHO, 2020

KEY ACTIONS	
□ Develop supply and distribution strategies for medicines and other health commodities that may be in short supply or are likely to be in high demand, taking into account safety and security.	
□ Adapt replenishment procedures to avoid community shortages, limiting facility encounters through multi-month dispensing, if supplies permit	
□ As supply levels allow, consider pre-positioning a buffer supply of at least a 1 month (and ideally longer) of essential resources for community-level service delivery. Designate resources specifically for use by the community health workforce, and anticipate increased resource needs.	
□ Coordinate the assessment, ordering and distribution of essential medicines, supplies (including PPE) and equipment with partners and community stakeholders.	
□ Ensure that pharmacies, health posts and other relevant public and private community-based entities are included in capacity assessments for the production and distribution of essential resources.	
□ Ensure that community-based pathways for medicine stock and distribution are included in electronic systems for order management, assessments and planning, if possible.	
□ For those making or accepting deliveries and when dispensing medicine or supplies, avoid excessive contact inside a health facility; for patients with chronic conditions, schedule medicine pick up via text (SMS) message or phone and maintain distance between patients while they wait.	
□ Consider using reverse logistics to reposition supplies based on the transmission scenario and feasibility in the local context.	

Although countries are beginning to lift social restrictions and are attempting societal reintegration in stages, the clinical and immunologic endpoints of the COVID-19 pandemic remain largely unknown. The course of the outbreak is likely to wax and wane, and thus the strategic response will need to be vigorous and dynamic. A taskforce dedicated to maintaining equitable access to contraception will be equipped to address health equity in any future crisis that may arise, providing a sustained benefit.

Access to contraception is a vital component of health care. Over the last 20 years, increasing contraceptive use in developing countries has reduced the number of maternal deaths by 40% via reduction in unintended pregnancy. Limited contraception access during the COVID-19 pandemic has the potential to reverse this progress. Although the full scope of the impact of the pandemic is not yet known, it is clear that negative impacts will disproportionately affect developing countries and marginalized communities, exacerbating worldwide sexual and reproductive health and justice inequities.

Although the unprecedented pandemic of COVID-19 has caused worldwide devastation, amidst the turmoil is an opportunity to develop a sustainable solution to the less overt yet alarming crisis of limited contraception provisioning.
